# A predictive model of macrosomic birth based upon real-world clinical data from pregnant women

**DOI:** 10.1186/s12884-022-04981-9

**Published:** 2022-08-18

**Authors:** Gao Jing, Shi Huwei, Chen Chao, Chen Lei, Wang Ping, Xiao Zhongzhou, Yang Sen, Chen Jiayuan, Chen Ruiyao, Lu Lu, Luo Shuqing, Yang Kaixiang, Xu Jie, Cheng Weiwei

**Affiliations:** 1 International Peace Maternity and Child Health Hospital, School of Medicine, Shanghai Jiao Tong University, No. 910 Hengshan Road, Shanghai, 200030 China; 2 Shanghai Key Laboratory of Embryo Original Disease, Shanghai, 200040 China; 3 Shanghai Municipal Key Clinical Specialty, Shanghai, 200030 China; 4 Shanghai Artificial Intelligence Laboratory, Shanghai, 200030 China; 5 The Second Affiliated Hospital of Nanjing Medical University, Nanjing, 210003 Jiangsu China

**Keywords:** Macrosomia, Prediction model, Nomogram, Clinical data

## Abstract

**Background:**

Fetal macrosomia is associated with an increased risk of several maternal and newborn complications. Antenatal predication of fetal macrosomia remains challenging. We aimed to develop a nomogram model for the prediction of macrosomia using real-world clinical data to improve the sensitivity and specificity of macrosomia prediction.

**Methods:**

In the present study, we performed a retrospective, observational study based on 13,403 medical records of pregnant women who delivered singleton infants at a tertiary hospital in Shanghai from 1 January 2018 through 31 December 2019. We split the original dataset into a training set (*n* = 9382) and a validation set (*n* = 4021) at a 7:3 ratio to generate and validate our model. The candidate variables, including maternal characteristics, laboratory tests, and sonographic parameters were compared between the two groups. A univariate and multivariate logistic regression was carried out to explore the independent risk factors for macrosomia in pregnant women. Thus, the regression model was adopted to establish a nomogram to predict the risk of macrosomia. Nomogram performance was determined by discrimination and calibration metrics. All the statistical analysis was analyzed using R software.

**Results:**

We compared the differences between the macrosomic and non-macrosomic groups within the training set and found 16 independent risk factors for macrosomia (*P* < 0.05), including biparietal diameter (BPD), head circumference (HC), femur length (FL), amniotic fluid index (AFI) at the last prenatal examination, pre-pregnancy body mass index (BMI), and triglycerides (TG). Values for the areas under the curve (AUC) for the nomogram model were 0.917 (95% CI, 0.908–0.927) and 0.910 (95% CI, 0.894–0.927) in the training set and validation set, respectively. The internal and external validation of the nomogram demonstrated favorable calibration as well as discriminatory capability of the model.

**Conclusions:**

Our model has precise discrimination and calibration capabilities, which can help clinical healthcare staff accurately predict macrosomia in pregnant women.

**Supplementary Information:**

The online version contains supplementary material available at 10.1186/s12884-022-04981-9.

## Background

Macrosomia refers to a birth weight that reaches 4000 g. It affects approximately 3–15% of pregnancies and frequently leads to adverse pregnancy outcomes such as shoulder dystocia, postpartum hemorrhage, and birth fractures [1]. A reliable prenatal predictor of macrosomia would therefore be of great significance for the optimization of clinical management and for improving maternal and infant outcomes.

Fetal weight is traditionally estimated by abdominal examination or ultrasonographic evaluation in obstetric practice [2]. The accuracy of abdominal examination is influenced by obesity, uterine fibroids, the amount of amniotic fluid, and clinical experience, and as ultrasonography is a practical method used to estimate the fetal weight (EFW), the post-test probability of identifying macrosomia varies from 15 to 79% with sonographic EFW [3]. This accuracy is significantly diminished with macrosomic infants, and low accuracy limits the predictive value of ultrasonograms.

Various predictive models based upon traditional statistical formulas or machine learning algorithms have been applied to the prediction of macrosomia in recent years. Daisuke et al. [4] developed an integer scoring system for excluding macrosomia using only maternal physical examination without sonographic information, and Wang et al. [5] constructed a random forest model that involved extra-pelvic measurement information and achieved improved sensitivity, specificity, and area under the receiver operating characteristic (ROC) curve (at 91.7, 91.7, and 95.3%, respectively). While the current predictive models obtained a higher degree of accuracy than the ultrasonographic and maternal abdominal evaluative methods, the models did not address comprehensive variables relevant to risk factors of macrosomia, and this might have weakened the overall accuracy of these models. Unusual indicators (e.g., carnitine metabolism or fetal soft tissue) were selected in some of the previous models, but these indicators are not always available, which is troubling to clinicians. Some models are exclusive in their predictions within a population of women with gestational diabetes mellitus (GDM) but neglect predictions in individuals without GDM [6]. Based on the aforementioned data, the use of comprehensive and readily available predictors—as well as the expansion of the study population—may augment the application and precision of the model.

In this study, we aimed to develop a more accurate, applicable, and stable model to predict the risk of macrosomia and employed a retrospective analysis of common clinical data that encompassed maternal characteristics, laboratory tests, and sonographic parameters in a large cohort of pregnant women. We believe that our study will provide a reference for the development of macrosomia prevention and appropriate intervention strategies.

## Methods

### Study population

**Fig. 1:**
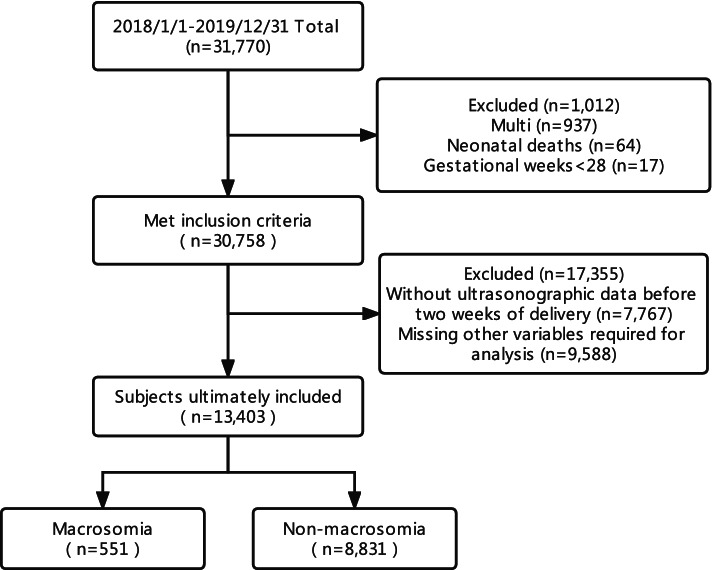
Chart illustrating patient flow in the present study

In this retrospective study, we extracted data from the digital medical records system of the International Peace Maternity and Child Health Hospital between 1 January 2018 and 31 December 2019. The inclusion criteria were as follows: (1) singleton pregnancy; (2) gestational weeks ≥28; (3) a normal pregnancy outcome (no stillbirths, neonatal deaths, or severe fetal malformations). After data screening, a total of 13,403 subjects were included in our study (Fig. 1).

### Data collection and variables included for analysis

We searched for variables of macrosomia that were reported in studies or systematic reviews, can be easily ascertained in different setting with various clinical experience, and are part of the routine examination during pregnancy. In this retrospective study, we collected maternal data that included demographics, clinical characteristics, laboratory tests, and fetal B-ultrasonographic examination. The extreme and error values of the measurement data were cleaned and the categorical data were normalized and coded.

At the first antenatal visit between 9 and 13 weeks of gestation, we collected data on the mother’s and husband’s demographic characteristics, medical history, and reproductive history. Maternal height, weight, gravity, parity, educational level, and basal blood pressure (systolic blood pressure, SBP; diastolic blood pressure, DBP) were recorded via face-to-face interviews. The pre-pregnancy body mass index (pre-pregnancy BMI) was calculated by dividing the pre-pregnancy weight (kg) by the pre-pregnancy height (m^2^), and numbers were divided into four levels: < 18.5 kg/m^2^ for underweight, 18.5–24.9 kg/m^2^ for normal weight, 25.0–29.9 kg/m^2^ for overweight, and 30 kg/m^2^ for obesity. Gestational weight gain (GWG) during pregnancy was determined by subtracting pre-pregnancy weight from the woman’s weight at her last prenatal examination. Appropriate gestational weight gain was stated as 12.5 kg to 18 kg for underweight, 11.5 kg to 16.0 kg for normal weight, 7 kg to 11.5 kg for overweight, and 5 kg to 9 kg for obesity according to the recommendations of the 2009 Institute of Medicine (IOM) guidelines categorized by pre-pregnancy BMI for each woman, and below or above the interval range was defined as insufficient or excessive weight gain [7].

We computed gestational age from the first day of the last menstrual period or the dating ultrasonographic scan performed prior to 20 weeks of pregnancy. Maternal fasting lipid serum samples were obtained in the first trimester (between nine and 14 weeks), collected in 10-mL vacutainer tubes, and centrifuged. Laboratory indices included triglycerides (TG), total cholesterol (TC), high-density lipoprotein (HDL), and low-density lipoprotein (LDL). The glucose index was obtained from a 75-g oral glucose tolerance test (OGTT) between gestational weeks 24 and 28—including fasting plasma glucose (FPG), one-hour glucose (GLU-1H), and two-hour glucose (GLU-2H), and hemoglobin (HbA1c). Routine sonographic evaluations performed by experienced doctors included fetal abdominal circumference (AC), biparietal diameter (BPD), head circumference (HC), humerus length (HL), femur length (FL), transverse trunk diameter (TTD), anteroposterior trunk diameter (APTD), and amniotic fluid index (AFI) at the last prenatal examination. The occurrence of macrosomia was the primary outcome used in this study. Shortly after birth, the newborns were weighed, their weights were recorded by medical staff, and those neonates with birth weights ≥4000 g were defined as manifesting macrosomia.

### Statistical analysis

We performed data analysis using R software version 4.1.2 (2021-11-01). Preliminary statistical analyzes including the Kolmogorov-Smirnov test [8] and Q-Q plots were performed to assess whether the data followed a normal distribution. Medians (and interquartile ranges, IQR) were used for continuous variables and counts and percentages for categorical variables. The Wilcoxon rank-sum test was employed for comparisons of continuous variables between groups, and the Chi-squared and Fisher’s exact probability tests were used for categorical variables, as appropriate. Differences were considered significant when they showed a *p*-value < 0.05.

The original dataset was randomly allocated to training and validation sets at a 7:3 ratio. A univariate logistic regression analysis was first performed to assess each variable’s significance separately. Any variables having a significant univariate test at a 0.05 level were selected as candidates for the following multivariate analysis. After the initial variable selection in the univariate analysis, multivariate logistic regression with a backward stepwise method was exploited within the training set to determine the risk factors associated with macrosomia. All variables screened by the backward stepwise algorithm would be included in the final model. Odds ratios and their corresponding 95% confidence intervals were then calculated for each independent variable. In addition, we employed ROC_AUC, sensitivity and specificity as our model evaluation metrics. To appraise the prediction capability of the logistic model and its fitness, we used the Hosmer and Lemeshow test and calculated the areas under the receiver operating characteristic curve (AUC). Multicollinearity was also tested on the final model by accessing the value of variance inflation factor (VIF). A nomogram model was then created based upon the final logistic regression model, and the nomogram model was validated by measuring discrimination and calibration curves both internally (training set) and externally (validation set). We assessed discrimination between observed and predicted outcomes using the metrics of ROC_AUC.

## Results

### Demographic and medical characteristics

The data from a total of 13,403 pregnant women were entered into our analysis. The original dataset was split into a training set (*n* = 9382) and a validation set (*n* = 4021), and we then compared the differences between the macrosomic and non-macrosomic groups within the training set. The mean birth weight of newborns in this study was 3345.9 g; 6893 (51%) of the neonates were male, and 6510 (49%) were female, with a macrosomia prevalence of 5.7%.

The demographic information is summarized in Table 1 and indicates that the BMI, gestational age (GA), SBP, and DBP were significantly higher in the macrosomic group than in the non-macrosomic group. Compared with the non-macrosomic group, the macrosomic group exhibited a significantly elevated percentage of excessive gestational weight gain (62% vs. 34%, *p* < 0.05) and showed a significantly reduced proportion of individuals with an educational level above a bachelor’s degree for both women (17% vs. 23%, *p* < 0.05) and their partners (22% vs. 25%, *p* < 0.05).

**Table 1:** Baseline characteristics of the study groups

Characteristic	Macrosomia^a^ ***n*** = 551	Non-macrosomia^a^ ***n*** = 8831	***p***-value^*^
BMI	22.2 (20.2, 24.2)	20.7 (19.2, 22.6)	< 0.001
Gestational age (GA)	39.5 (39.0, 40.3)	39.1 (38.4, 39.6)	< 0.001
Gravidity	2.0 (1.0, 3.0)	2.0 (1.0, 2.0)	< 0.001
Parity	1.0 (1.0, 2.0)	1.0 (1.0, 2.0)	0.074
SBP	112 (104, 121)	110 (102, 119)	< 0.001
DBP	69 (63, 76)	68 (62, 75)	0.029
Age			0.087
≥ 35	418 (76%)	6971 (79%)	
< 35	133 (24%)	1860 (21%)	
Husband age			0.021
≥ 35	354 (64%)	6089 (69%)	
< 35	197 (36%)	2742 (31%)	
Educational level			< 0.001
Bachelor’s degree	293 (53%)	4728 (54%)	
Above bachelor’s	93 (17%)	,2020 (23%)	
Below bachelor’s	165 (30%)	2083 (24%)	
Husband’s educational level			0.017
Bachelor’s degree	286 (52%)	4762 (54%)	
Above bachelor’s	121 (22%)	2198 (25%)	
Below bachelor’s	144 (26%)	1871 (21%)	
Conception			0.12
Natural conception	498 (90%)	8144 (92%)	
Assisted reproduction	53 (9.6%)	687 (7.8%)	
GWG			< 0.001
Optimal	165 (30%)	3754 (43%)	
Inadequate	44 (8.0%)	2064 (23%)	
Excessive	342 (62%)	3013 (34%)	
Smoking-tobacco use			0.8
No	547 (99%)	8771 (99%)	
Yes	4 (0.7%)	60 (0.7%)	
Alcohol use			0.2
No	530 (96%)	8571 (97%)	
Yes	21 (3.8%)	260 (2.9%)	
Family history of diabetes or hypertension			0.6
No	422 (77%)	6693 (76%)	
Yes	123 (22%)	1996 (23%)	
Unknown	(1.1%)	142 (1.6%)	

^a^ Median (IQR); n (%)

^*^Wilcoxon rank-sum test; Pearson’s Chi-squared test; Fisher’s exact probability test

Table 2 depicts the medical characteristics, including ultrasonographic and clinical laboratory test results. BPD, HC, FL, HL, TTD, APTD, AC, and AFI showed significantly higher median values in the macrosomic group compared to the non-macrosomic group. In terms of clinical laboratory findings, FPG, GLU-1H, GLU-2H, and TG in the macrosomic group were significantly augmented relative to the non-macrosomic group, while HDL was lower than in the non-macrosomic group. TC (*p* = 0.6) and LDL (*p* = 0.4) did not differ between groups.

**Table 2:** Medical characteristics of the study groups

Characteristic	Macrosomia^a^ ***n*** = 551	Non-macrosomia^a^ ***n*** = 8831	***p***-value^*^
BPD	97.0 (95.0, 99.0)	94.0 (92.0, 96.0)	< 0.001
HC	332 (324, 338)	320 (313, 328)	< 0.001
FL	72.0 (70.0, 73.0)	69.0 (67.0, 71.0)	< 0.001
HL	63.0 (62.0, 64.0)	60.0 (59.0, 62.0)	< 0.001
TTD	108 (105, 112)	101 (97, 105)	< 0.001
APTD	110 (106, 114)	103 (99, 107)	< 0.001
AC	342 (334, 351)	320 (309, 331)	< 0.001
AFI	131 (108, 157)	120 (102, 142)	< 0.001
FPG	4.30 (4.04, 4.60)	4.20 (3.96, 4.46)	< 0.001
GLU-1H	7.92 (6.93, 9.01)	7.61 (6.67, 8.74)	< 0.001
GLU-2H	6.69 (5.86, 7.61)	6.41 (5.62, 7.35)	< 0.001
HbA1c	5.00 (4.80, 5.20)	5.00 (4.80, 5.10)	< 0.001
TG	1.39 (1.09, 1.74)	1.28 (1.02, 1.62)	< 0.001
TC	4.44 (3.97, 4.89)	4.44 (4.00, 4.92)	0.6
HDL	1.85 (1.59, 2.15)	1.94 (1.68, 2.21)	< 0.001
LDL	2.51 (2.12, 2.97)	2.50 (2.12, 2.94)	0.4

^a^ Median (IQR)

^*^ Wilcoxon rank-sum test

### Regression analysis and risk factors for macrosomia

Our multivariate regression analysis of factors associated with macrosomia is shown in Table 3. The model was established with macrosomia as the outcome variable and twenty-four significant indices in the univariate analysis as independent variables using backward stepwise regression. Sixteen predictors were included in the final model: E educational level, GWG, fetal sex, gravidity (GNUM), BMI, GA, AC, BPD, HC, FL, HL, TTD, AFI, FPG, GLU-1H, and TG. The results of the Hosmer and Lemeshow test provide a *p*-value of 0.15, that is greater than 0.05, indicating no evidence of poor fit and our model is correctly specified. We also analyzed multicollinearity, with all indices showing a VIF of less than 3, and thus, we had no issue with collinearity.

**Table 3:** Factors associated with macrosomia among women at the international peace maternity and child health hospital (*n* = 9382)

Characteristic	OR^a^	95% CI^b^	***p***-value
Educational level			
Bachelor’s degree	–	–	
Above bachelor’s	0.75	(0.56, 0.98)	0.037
Below bachelor’s	1.19	(0.93, 1.51)	0.2
GWG			
Optimal	–	–	
Inadequate	0.51	(0.34, 0.74)	< 0.001
Excessive	1.59	(1.27, 2.00)	< 0.001
Fetal Sex			
Female	–	–	
Male	1.67	(1.34, 2.08)	< 0.001
GNUM	1.14	(1.04, 1.24)	0.005
BMI	1.07	(1.03, 1.11)	< 0.001
GA	1.22	(1.08, 1.37)	0.001
AC	1.08	(1.06, 1.09)	< 0.001
BPD	1.08	(1.03, 1.14)	0.003
HC	1.03	(1.01, 1.04)	< 0.001
FL	1.08	(1.01, 1.15)	0.023
HL	1.19	(1.12, 1.26)	< 0.001
TTD	1.02	(0.99, 1.05)	0.12
AFI	1.01	(1.00, 1.01)	< 0.001
FPG	1.44	(1.11, 1.87)	0.006
GLU-1H	1.09	(1.01, 1.18)	0.030
TG	1.17	(0.97, 1.40)	0.093

^a^ *OR* Odds ratio

^b^ *CI* Confidence interval

### Nomogram construction and validation

**Fig. 2:**
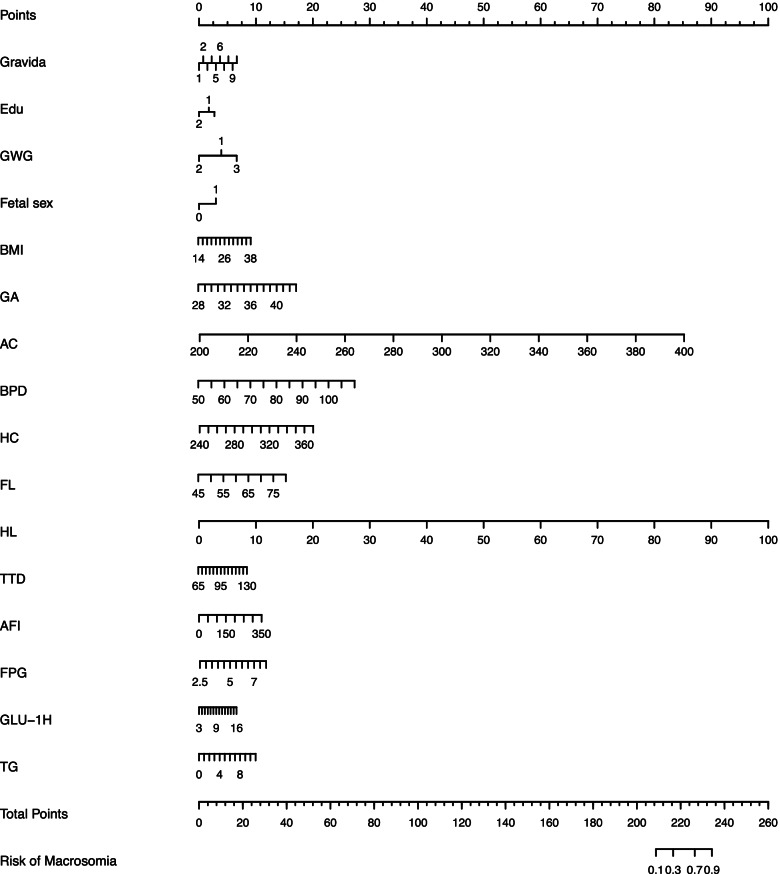
Nomogram model for predicting the risk of macrosomia. Nomogram model for predicting the risk of macrosomia using 16 predictors: Gravida, gravidity; Edu, educational level; GWG, gestational weight gain; fetal sex; BMI, body mass index; GA, number of gestational weeks; AC, abdominal circumference; BPD, biparietal diameter; HC, head circumference; FL, femur length; HL, humerus length; TTD, transverse trunk diameter; AFI, amniotic fluid index; FPG, fasting plasma glucose; GLU-1H, glucose at one-hour post-OGTT; TG, triglycerides

**Fig. 3:**
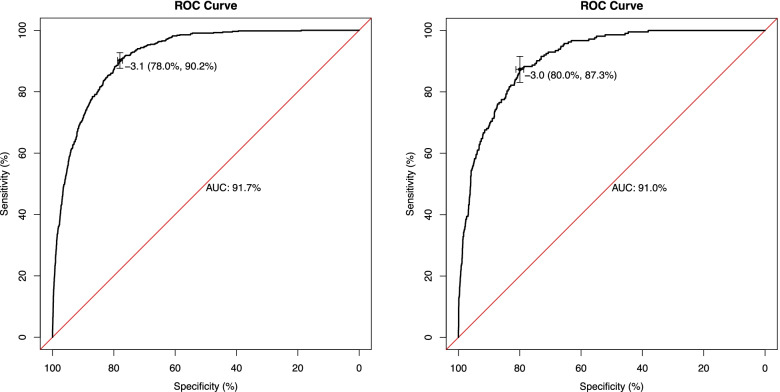
ROC curve of macrosomia. The ROC curve of macrosomia concerning its internal validation is shown in the left panel, and that for external validation is shown in the right panel

**Fig. 4:**
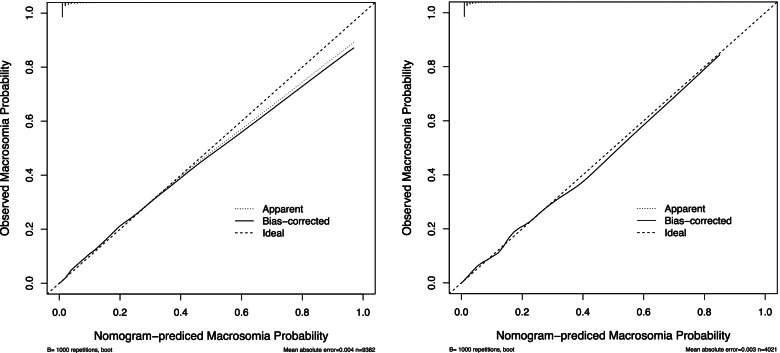
Calibration curve. The calibration curve for the internal validation of the nomogram model is shown in the left panel, and the calibration curve for external validation is shown in the right panel

We constructed a nomogram model based upon the 16 predictors noted above to predict the risk of macrosomia (Fig. 2), with each predictor given a point according to the characteristics of each woman; the total number of points was then calculated to obtain the risk of macrosomia. The model achieved satisfactory performance, obtaining a sensitivity of 0.898 and a specificity of 0.781 with the optimal probability threshold chosen. Additionally, the values for the AUCs of the nomogram model were 0.917 (95% CI, 0.908–0.927) and 0.910 (95% CI, 0.894–0.927) in the training set and validation set, respectively, indicating that the model was robust in its discriminative ability (Fig. 3). Both internal and external calibration curves also confirmed that there was a favorable concordance between the observed and predicted probabilities (Fig. 4).

## Discussion

In this single-center retrospective study, we developed and validated a nomogram model for the prediction of macrosomia among newborns and achieved satisfactory predictive effects based upon the clinical data from a large cohort of pregnant women.

Pregnancy is a complex process accompanied by substantial changes in sugar and lipid metabolism [9]. Considering that these indicators may be key factors that contribute to fetal weight changes, we combined the key indicators (including blood glucose and lipid parameters) with the general indicators (for example, demographic characteristics and fetal intuitive sonography image measurements) as variables in our model.

It has been proposed that maternal hyperglycemia leads to fetal hyperglycemia, stimulating maturation and hypertrophy of the fetal pancreas [9], and various studies have indicated that GDM constitutes one of the important factors affecting the onset and development of macrosomia [8, 10]. The results of our study revealed that the incidence of macrosomia was 6.38% (115/1802) for women with GDM and 5.65% (649/11,482) for women with non-GDM, numbers consistent with the previous studies [11]. Several authors have supported pregnant women’s blood glucose levels as strongly associated with the incidence of macrosomia, regardless of a diagnosis of GDM [12, 13]. We usually perform an OGTT on pregnant women between their 24th and 28th gestational weeks to diagnose GDM, as OGTT, FPG, GLU-1H, GLU-2H, and HBA1C are important indicators in the assessment of maternal blood glucose levels. Through multivariate analysis, fasting glucose and OGTT-1H were then utilized as indicators in our model.

Maternal serum lipids may comprise an important fuel in fetal overgrowth during the entire pregnancy [14]. Since Xue et al. hypothesized that elevated TG levels in early pregnancy but not in late pregnancy were crucial risk factors associated with the incidence of fetal macrosomia [15], we then chose the lipid index in early pregnancy as the variable we used for the prediction of macrosomia. When we herein collected four maternal lipid parameters (TC, TG, HDL, and LDL) in the first trimester (9th and 13th gestational weeks) of pregnancy, our results revealed that TGs showed high specificity in the prediction of macrosomia, and we, therefore, chose TGs as the predictive indicator in our model.

The most objective method currently employed to estimate fetal body weight is ultrasonographic (US) measurement, which encompasses over 30 different formulas for the US estimates to predict newborn birth weight [16–18], with the most widely used being the Hadlock formula [19]. To generate sonographic fetal weight estimations with a lower error margin, many formulas have reflected disparate parameters of the fetus (fetal abdominal fat layer [20], shoulder soft-tissue thickness [21], biacromial diameter [22]), and some have even entailed 3D sonographic measurements [23]. Although such formulas and novel predictors may improve the accuracy of US evaluation, they nevertheless increase technical difficulty and sonication time. Considering the availability of predictors, we used B-ultrasonography 2 weeks prior to delivery to assess the predictive capability of our model.

The nomogram model can transform the cumbersome regression equation into a visually legible graph that is both convenient and rapid in its practical application [24]. Each predictor is given a point according to the woman’s characteristics, and the point total is then calculated to obtain the risk of macrosomia. Mazouni et al. developed a nomogram to predict macrosomia based on maternal demographic characteristics and US variables, with the model achieving moderate predictive ability at an AUC of 0.850 [25]. These authors’ sample size was, however, quite small and dismissed the influence of maternal metabolism. Although Sun et al. established a nomogram model combined with carnitine-related metabolic variables for predicting macrosomia in pregnant women with GDM [26], carnitine metabolism is not routinely used in the clinical setting, restricting its application. Shigemi et al. created a scoring system based upon the significant predictors of macrosomia without sonographic information [4], and their system exhibited a high negative predictive value of 0.996–1.000, while the positive predictive value for screening macrosomia was extremely low (0.003). Zou et al. [27] and Kang et al. [28] published models that could only be applied to women with GDM rather than to all pregnant women, and Ye et al. used ensemble methods (one comprising a machine-learning algorithm) to improve the prediction of fetal macrosomia [18]. Unfortunately, ensemble methods are cumbersome and limited in their practicability. In contrast, our model was applied to the entire population of pregnant women and displayed many advantages. First, the precision of our model met or exceeded the optimal predictive levels recorded in the literature [25, 27]. The areas under the ROC curves (AUCs) for the internal and external validation of our model were 91.7 and 91.0, respectively. In addition, using our model, we selected alternative but still routine clinical data that were easily accessible and relatively comprehensive. Macrosomia risk factors can be classified into three components: maternal characteristics, metabolic parameters, and US measurements. However, as most models only incorporate some of these three risk factors to predict the incidence of macrosomia, we posit that our predictive model is more generalizable, precise, and clinically suitable.

Our nomogram model could be a practical tool for clinical work. Once the model shows the possibility of macrosomia, suggesting that pregnant women might be in an over-nutrition condition and need strictly controlled weight gain by lifestyle, diet, exercise. Meanwhile, doctors enhance close monitoring and supervision on them. Nowadays, over-estimated fetal weight could result in over-classification of fetuses as macrosomic with unnecessary cesarean deliveries, under-estimated fetal weight could also pose a risk of dystocia or even stillbirth. Our model fits with the current strategy for precision medicine can guide the mode of delivery and provide assistance at birth. For example, if the model shows a high probability of macrosomia, we can arrange medical personnel, drugs and medical supplies ahead of time in order to prevent postpartum hemorrhage, shoulder dystocia, severe perineal lacerations etal actively. All in all, our model judges the macrosomia accurately and covers the clinical pregnancy management during the antenatal, intrapartum and postpartum periods. Significantly, our model will be a strong aidarm to enhance doctors and midwives’ decision confidence, as well as bring lower anxiety of pregnant women due to the uncertainty of their fetal weight. There were some limitations to the present study. First, we are a single obstetric hospital that principally covers low-to-moderate-risk pregnant women, and this cohort may not fully represent all obstetric practices in the community. Second, this was a retrospective study that lacked the validation of relevant variables, thus slightly reducing its overall credibility; in the next phase, we will include the relevant variables and appropriate influencing factors and initiate a prospective study. Finally, the occurrence of macrosomia is affected by many factors, including the environment. For example, some evidence suggests that exposure of pregnant women to air pollutants [29], such as PM_2.5_, NO_2_, and O_3_, and passive smoking may also increase the fetal risk for macrosomia. We herein ignored the influences of environmental effects, lifestyle, work stress, and social relationships.

## Conclusions

In summary, our proposed predictive nomogram model can be used effectively to prognosticate the incidence of macrosomia. The highly predictive sensitivity and specificity of our model can thus aid clinicians in reducing adverse pregnancy outcomes. In the future, we will convert the nomogram model into an electronic medical records system or mobile application for every pregnant woman to expand the potential value of this predictive model.

## Supplementary Information

**Additional file 1: Supplemental Table.** Meaning of all the 24 variables.

## Data Availability

The datasets analyzed during the current study are not publicly available due to the metadata containing information that could compromise the patients but are available from the corresponding author on reasonable request.

## Abbreviations

AC: Abdominal circumference
AFI: Amniotic fluid index
APTD: Anteroposterior trunk diameter
AUC: Area under the curve
BMI: Body mass index
BPD: Biparietal diameter
DBP: Diastolic Blood Pressure
EFW: Estimating fetal weight
FL: Femur length
FPG: Fasting plasma glucose
GA: Gestational age
GDM: Gestational diabetes mellitus
GLU-1H: 1-hour glucose
GLU-2H: 2-hour glucose
GNUM: Gravidity
GWG: Gestational weight gain
HbA1c: Hemoglobin
HC: Head circumference
HDL: High-density lipoprotein
HL: Humerus length
IOM: Institute of Medicine
IQR: Interquartile ranges
LDL: Low-density lipoprotein
OGTT: Oral glucose tolerance test
ROC: Receiver operating characteristic
SBP: Systolic Blood Pressure
TC: Total cholesterol
TG: Triglycerides
TTD: Transverse trunk diameter
VIF: Variance inflation factor

## References

[1] Barth Jr W H, Jackson R. Macrosomia ACOG Practice Bulletin, Number 216. Obstet Gynecol. 2020;135(1):E18–E35. 10.1097/AOG.0000000000003606 31856124

[2] Nguyen MT, Ouzounian JG (2021). Evaluation and management of fetal macrosomia. Obstet Gynecol Clin N Am.

[3] Melamed N, Yogev Y, Meizner I, Mashiach R, Pardo J, Ben-Haroush A (2011). Prediction of fetal macrosomia: effect of sonographic fetal weight-estimation model and threshold used. Ultrasound Obstet Gynecol.

[4] Shigemi D, Yamaguchi S, Aso S, Yasunaga H (2019). Predictive model for macrosomia using maternal parameters without sonography information. J Matern Fetal Neonatal Med.

[5] Wang F, Wang Y, Ji X, Wang Z (2022). Effective macrosomia prediction using random forest algorithm. Int J Environ Res Public Health.

[6] Hua XG, Jiang W, Hu R (2020). Large for gestational age and macrosomia in pregnancies without gestational diabetes mellitus. J Matern Fetal Neonatal Med.

[7] To IOMU, Guidelines RIPW (2009). Weight gain during pregnancy: reexamining the guidelines.

[8] Scholtens DM, Kuang A, Lowe LP (2019). Hyperglycemia and adverse pregnancy outcome follow-up study (HAPO FUS): maternal glycemia and childhood glucose metabolism. Diabetes Care.

[9] Langer O (2000). Fetal macrosomia: Etiologic factors. Clin Obstet Gynecol.

[10] Farrar D, Simmonds M, Bryant M (2016). Hyperglycaemia and risk of adverse perinatal outcomes: systematic review and meta-analysis. BMJ.

[11] Li G, Kong L, Li Z (2014). Prevalence of macrosomia and its risk factors in China: a multicentre survey based on birth data involving 101,723 singleton term infants. Paediatr Perinat Epidemiol.

[12] Metzger BE, Lowe LP, Dyer AR (2008). Hyperglycemia and adverse pregnancy outcomes. N Engl J Med.

[13] James-Todd TM, Karumanchi SA, Hibert EL (2013). Gestational age, infant birth weight, and subsequent risk of type 2 diabetes in mothers: Nurses' health study II. Prev Chronic Dis.

[14] Nasioudis D, Doulaveris G, Kanninen TT (2019). Dyslipidemia in pregnancy and maternal-fetal outcome. Minerva Ginecol.

[15] Xue RH, Wu DD, Zhou CL (2021). Association of high maternal triglyceride levels early and late in pregnancy with adverse outcomes: a retrospective cohort study. J Clin Lipidol.

[16] Cesnaite G, Domza G, Ramasauskaite D, Volochovic J (2020). The accuracy of 22 fetal weight estimation formulas in diabetic pregnancies. Fetal Diagn Ther.

[17] Stirnemann J, Villar J, Salomon LJ (2017). International estimated fetal weight standards of the INTERGROWTH-21(st) project. Ultrasound Obstet Gynecol.

[18] Ye S, Zhang H, Shi F, et al. Ensemble learning to improve the prediction of fetal macrosomia and large-for-gestational age. J Clin Med. 2020;9(2):380. 10.3390/jcm9020380 PMC7074295 32023935

[19] Hadlock FP, Harrist RB, Sharman RS, Deter RL, Park SK (1985). Estimation of fetal weight with the use of head, body, and femur measurements--a prospective study. Am J Obstet Gynecol.

[20] Elessawy M, Harders C, Kleinwechter H (2017). Measurement and evaluation of fetal fat layer in the prediction of fetal macrosomia in pregnancies complicated by gestational diabetes. Arch Gynecol Obstet.

[21] Aliyeva M, Aydin S (2021). Use of ultrasound fetal shoulder soft tissue thickness measurement in estimation of fetal weight. J Obstet Gynaecol Res.

[22] Youssef A, Amin AF, Khalaf M, Khalaf MS, Ali MK, Abbas AM (2019). Fetal biacromial diameter as a new ultrasound measure for prediction of macrosomia in term pregnancy: a prospective observational study. J Matern Fetal Neonatal Med.

[23] Mazzone E, Dall'Asta A, Kiener A (2019). Prediction of fetal macrosomia using two-dimensional and three-dimensional ultrasound. Eur J Obstet Gynecol Reprod Biol.

[24] Park SY (2018). Nomogram: an analogue tool to deliver digital knowledge. J Thorac Cardiovasc Surg.

[25] Mazouni C, Rouzier R, Ledu R, Heckenroth H, Guidicelli B, Gamerre M (2007). Development and internal validation of a nomogram to predict macrosomia. Ultrasound Obstet Gynecol.

[26] Sun M, Zhao B, He S (2020). The alteration of carnitine metabolism in second trimester in GDM and a nomogram for predicting macrosomia. J Diabetes Res.

[27] Zou Y, Zhang Y, Yin Z, Wei L, Lv B, Wu Y (2021). Establishment of a nomogram model to predict macrosomia in pregnant women with gestational diabetes mellitus. BMC Pregnancy Childbirth.

[28] Kang X, Liang Y, Wang S (2021). Prediction model comparison for gestational diabetes mellitus with macrosomia based on risk factor investigation. J Matern Fetal Neonatal Med.

[29] Shang L, Yang L, Yang W (2021). Prenatal exposure to air pollution and the risk of macrosomia: identifying windows of susceptibility. Sci Total Environ.

