# Supplementary material for: A predictive model of macrosomic birth based upon real-world clinical data from pregnant women
Source: BMC Pregnancy Childbirth. 2022 Aug 18;22:651. doi: 10.1186/s12884-022-04981-9 (PMC9386989; doi:10.1186/s12884-022-04981-9)
Supplement: Supplementary file 1 — Additional file 1: Supplemental Table. Meaning of all the 24 variables. [file 12884_2022_4981_MOESM1_ESM.docx]

**Supplemental Material**

**Supplemental Table Meaning of all the 24 variables**

| **variable** | **variable meaning** |
| --- | --- |
| Edu  Hus edu  Hus age  Gravida  Fetal sex  GWG  BMI  SBP  DBP  AC  BPD  HC  FL  HL  TTD  APTD  HBA1C  FPG  GLU-2H  GLU-1H  TG  HDL  AFI | Educational level  Husband’s educational level  Husband’s age  gravidity  Fetal sex  gestational weight gain  pre-pregnancy body mass index  systolic blood pressure  diastolic blood pressure  abdominal circumference  biparietal diameter  head circumference  femur length  humerus length  transverse trunk diameter  anteroposterior trunk diameter  hemoglobin  fasting plasma glucose  two-hour glucose  one-hour glucose  triglycerides  high-density lipoprotein  Amniotic Fluid Indexes |
| GA | Gestational age |
